# Global potential distribution of *Paeonia lactiflora* and its climate-driven shifts: insights from an enhanced MaxEnt model integrating soil and solar radiation variables

**DOI:** 10.3389/fpls.2026.1756429

**Published:** 2026-02-13

**Authors:** Jinyu Cai, Shu Wang, Zheng Fan, Rongchun Han, Xiaohui Tong

**Affiliations:** 1 School of Pharmacy, Anhui University of Chinese Medicine, Hefei, China; 2 Department of Medical Affairs, The Seventh Affiliated Hospital of Anhui University of Chinese Medicine, Taihe, China; 3 School of Life Sciences, Anhui University of Chinese Medicine, Hefei, China

**Keywords:** climate change, environmental factors, MaxEnt, *Paeonia lactiflora*, potential distribution, species distribution model

## Abstract

*Paeonia lactiflora* Pall. is a globally important medicinal perennial whose habitat suitability remains poorly known beyond China. Using an enhanced MaxEnt model integrating 45 climatic, soil, and solar radiation variables, we predicted its current and future global distribution based on 833 spatially thinned occurrence records and 12 low-collinearity predictors. The model performed excellently (test AUC = 0.945 ± 0.001; TSS = 0.762 ± 0.018). Precipitation of the warmest quarter (bio18), mean temperature of the coldest quarter (bio11), temperature seasonality (bio4), and November solar radiation (srad11) were the dominant drivers. Currently, total suitable habitat is centered in East Asia, central Europe, and northeastern/midwestern USA. All future scenarios (SSP2-4.5 and SSP5-8.5, 2041–2060 and 2061–2080) project about 25–45% expansion of total suitable area, accompanied by a consistent northeastward centroid shift of highly suitable habitat (up to ~1,234 km under SSP5-8.5 2061–2080). Late-century high-emission conditions cause localized contraction of core habitat in southern margins. *P. lactiflora* is likely to benefit from moderate warming, but high-emission pathways will drive major reorganization and degradation of traditional production areas, necessitating strengthened conservation in current strongholds and proactive planning in emerging northern regions.

## 1 Introduction

*Paeonia lactiflora* Pall., a perennial herbaceous species in the family Paeoniaceae, has been used medicinally for over two millennia (Shagjjava et al., 2016). Its earliest recorded medicinal use appears in the “Prescriptions for Fifty-two Diseases,” a medical text unearthed from the Mawangdui Han Tomb in Changsha, China. For more than 2,000 years, *P. lactiflora* and its processed root products—Paeoniae Radix Alba and Paeoniae Radix Rubra—have played a central role in traditional Chinese medicine. According to the Chinese Pharmacopoeia, the primary authentic production regions (*dao-di areas*) are located in northeastern China, northern China, Shaanxi Province, and northern Gansu Province, although the species is now widely cultivated throughout the country.

*P. lactiflora* exhibits relatively strong environmental adaptability and typically occurs on grassy slopes, forest margins, and in thickets within temperate regions. Its growth and development are strongly influenced by environmental factors, particularly light availability, soil moisture, and temperature (Zhang et al., 2019). Suitable ecological conditions are essential for the accumulation of its bioactive compounds. Chemical studies have shown that *P. lactiflora* is rich in monoterpene glycosides (primarily paeoniflorin), tannins, flavonoids, polysaccharides, and other active constituents, which underpin its significant medicinal and economic value (He and Dai, 2011).

Despite its long history of clinical use and well-documented efficacy, the environmental factors driving the formation of its authentic production regions (*dao-di areas*) and the extent of genetic diversity in germplasm resources remain poorly understood (Huang, 2011). Moreover, wild populations of *P. lactiflora* are increasingly threatened by habitat loss and overharvesting, posing serious challenges to germplasm conservation and sustainable utilization (Yadav et al., 2024).

Species distribution models (SDMs) have become indispensable tools for assessing species’ geographic patterns and their responses to environmental change, with wide applications in conservation biology, medicinal plant resource management, and biogeography. Among the various SDM approaches, MaxEnt (maximum entropy) stands out for its robust performance when using presence-only data and relatively small sample sizes, making it particularly well-suited for studying threatened or poorly documented medicinal plants (Phillips et al., 2004). The algorithm is grounded in the principle of maximum entropy, which provides a statistically rigorous framework for generating predictive distributions from incomplete information.

Proposed by Jaynes (1957), the principle of maximum entropy states that, among all probability distributions consistent with the available constraints, the one with the highest entropy is the least biased and most appropriate (Shore and Johnson, 1980). In the context of species distribution modelling, entropy reflects predictive uncertainty: higher entropy corresponds to a more uniform (less environmentally constrained) predicted distribution, whereas lower entropy indicates stronger environmental filtering (Banavar et al., 2010).

Unlike many traditional modelling approaches that rely on restrictive assumptions about species–environment relationships (e.g., linearity or predefined response curves), MaxEnt imposes minimal *a priori* constraints. It uses only empirically derived relationships between known occurrence records and environmental predictors, avoiding the extrapolation of untested ecological assumptions. This assumption-lean, data-driven framework makes MaxEnt especially valuable for modelling poorly surveyed or narrowly distributed species, including many medicinal plants with limited occurrence data (Lissovsky and Dudov, 2021).

In practice, MaxEnt constructs a probability distribution over the study area that maximizes entropy (i.e., is as uniform as possible) while remaining consistent with the empirical constraints derived from known occurrences. For a given plant species, these constraints typically include the observed ranges and variances of environmental variables (e.g., temperature and precipitation) at presence localities (Elith et al., 2011). The resulting distribution is the least committed to unobservable patterns, effectively balancing ecological signal against sampling noise and bias.

A key strength of MaxEnt is its ability to capture nonlinear responses and interactions among predictors without assuming specific functional forms (e.g., quadratic or threshold responses). Because it relies solely on empirically supported constraints, the model naturally accommodates complex, non-additive relationships that are common in real species–environment associations (Halvorsen et al., 2016).

ArcGIS provides robust tools for spatial data processing, standardization, and visualization, making it widely used in species distribution modelling (Wong and Lee, 2005; Scott and Janikas, 2009). In this study, it served three primary functions: (1) pre-processing and formatting of occurrence records and environmental layers to meet MaxEnt input requirements; (2) derivation of additional topographic and bioclimatic variables; and (3) post-modelling visualization, including production of habitat suitability maps, calculation of suitable area, and centroid analysis of highly suitable regions (Yan et al., 2020; Zeng et al., 2021; Xu et al., 2024). This integrated GIS–MaxEnt workflow substantially streamlines data preparation, model implementation, and result interpretation.

Climate change is profoundly affecting the growth, phenology, and geographic distribution of plant species worldwide (Li et al., 2022). Although previous studies have used MaxEnt and Biomod2 to model the potential distribution of *P. lactiflora* within China (Bi et al., 2022; Wang et al., 2024), the species also maintains naturalized or cultivated populations in parts of East Asia, Europe, and North America (Shagjjava et al., 2016). To date, no study has assessed its global habitat suitability or projected climate-driven range shifts at a worldwide scale.

This study addresses these gaps by: (1) compiling a comprehensive global occurrence dataset; (2) incorporating a broader suite of environmental predictors, including soil properties and solar radiation in addition to climatic variables; and (3) evaluating both current suitability and future range dynamics under multiple shared socioeconomic pathways (SSPs) and time periods. These improvements are expected to yield more robust and biologically realistic predictions than regional models that rely solely on climatic data.

## 2 Materials and methods

### 2.1 Acquisition of global *P. lactiflora* geographic distribution data

Global occurrence records of *P. lactiflora* were downloaded from the Global Biodiversity Information Facility (GBIF.org (17 July 2025) GBIF Occurrence Download https://doi.org/10.15468/dl.vpzb7g). We retained only records with valid geographic coordinates collected between 1970 and 2025, yielding an initial set of 1,434 presence points. Records lacking coordinates, duplicate entries, or obvious georeferencing errors (e.g., located in the ocean or at coordinate origin 0,0) were removed during initial cleaning (Radosavljevic and Anderson, 2014).

**Figure 1:**
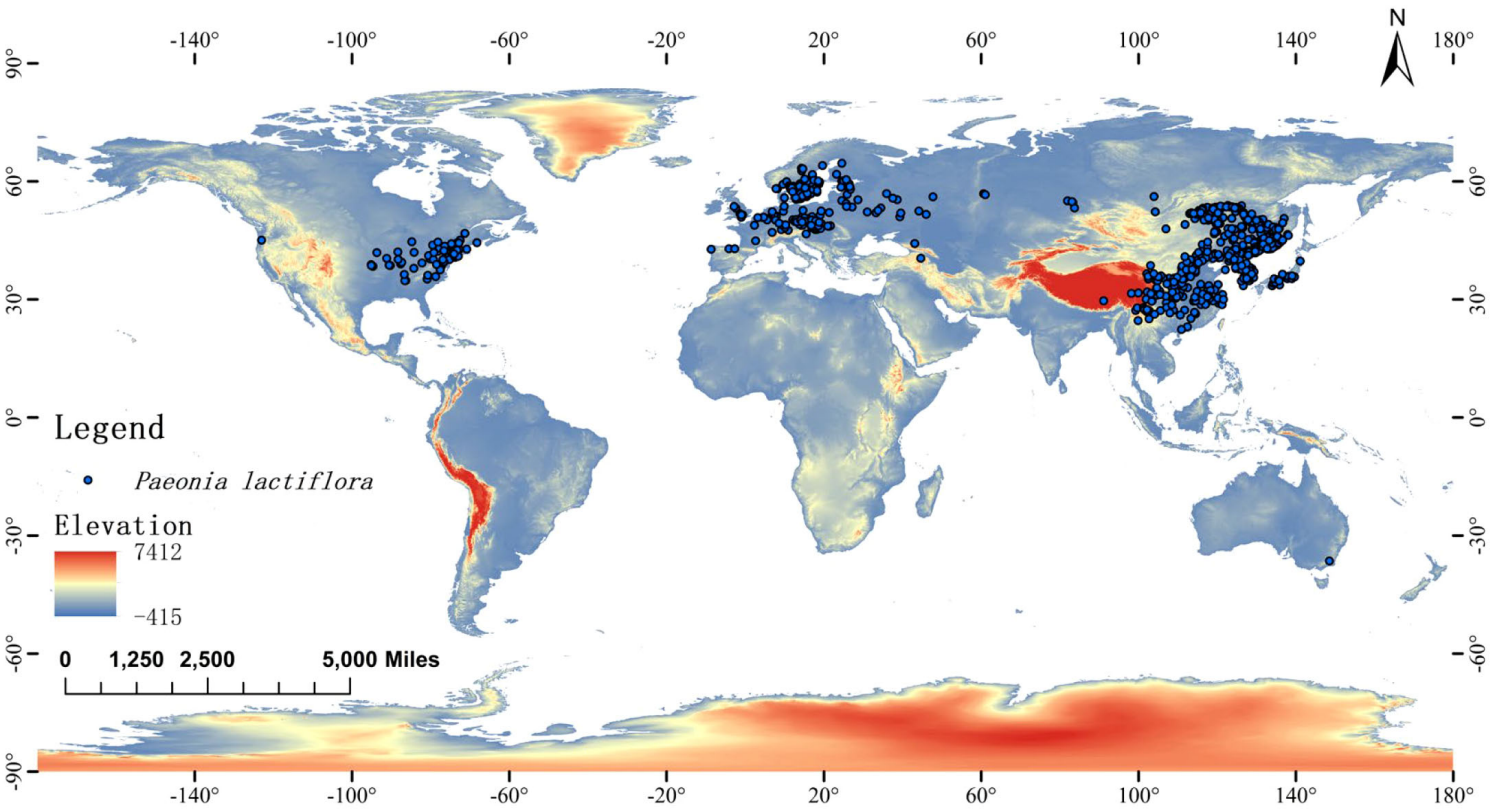
Distribution of *P. lactiflora*, with each blue dot indicating a recorded occurrence point.

To reduce spatial autocorrelation and sampling bias caused by clustered collections, the dataset was spatially thinned using the “Spatially Rarefy Occurrence Data” tool in SDMtoolbox 2.0 for ArcGIS (Isaac et al., 2020). A thinning distance of 20 km was applied, resulting in a final set of 833 spatially independent occurrence records (**Figure 1**). This thinning distance was chosen to approximate the spatial resolution of the environmental layers used (ca. 5 arc-min, ≈10 km at the equator) while retaining sufficient records for robust modelling.

### 2.2 Processing of environmental variable data

The distribution of herbaceous perennials in the Paeoniaceae is strongly governed by moisture availability, temperature extremes, solar radiation, and soil physical and chemical properties (Shagjjava et al., 2016). To capture these drivers at a global scale, we compiled an initial set of 45 environmental layers representing climatic, edaphic, and radiation conditions.

Current (1970–2000) bioclimatic variables (19 variables), monthly precipitation, solar radiation (srad), and shortwave radiation downward flux (swd) were obtained from WorldClim version 2.1 at 5 arc-minute resolution (~10 km at the equator) (Fick and Hijmans, 2017). Elevation was derived from the GMTED2010 dataset and resampled to the same grid. Soil data were extracted from the Harmonized World Soil Database v1.2 (HWSD). Soil layers were held constant across future periods, a standard assumption in global-scale SDM studies (Slessarev et al., 2019). This practice is justified because soil properties (e.g., texture, organic carbon, pH) form and change over centennial to millennial timescales through processes such as weathering, organic matter accumulation, and erosion, which are far slower than the decadal-to-centennial pace of projected climate change under CMIP6 scenarios (Meyer et al., 2025). High-resolution global soil data for future conditions are currently unavailable, and dynamic soil models are computationally intensive and not yet sufficiently validated for broad-scale projections (Ni and Vellend, 2024).

Nevertheless, this assumption introduces limitations. Soil properties may shift indirectly under prolonged climate change through altered vegetation dynamics, erosion rates, or microbial activity, potentially modifying habitat suitability in ways not captured here. In particular, in arid or high-latitude regions where soil formation is slow but climate-driven degradation (e.g., desertification or permafrost thaw) is rapid, future suitability could be overestimated or underestimated (Huang et al., 2017). Future refinements could incorporate dynamic soil–climate feedbacks or scenario-based soil projections when such data become available. To maintain consistency, future soil layers were assumed to remain unchanged, a standard practice in global-scale SDM projections given the slow rate of soil property change relative to climate. From the HWSD, we retained ten topsoil (0–30 cm) attributes known to influence root development and nutrient uptake in medicinal Peony species: drainage class, USDA texture class, sand fraction, clay fraction, pH (water), organic carbon, CaCO_3_ content, electrical conductivity, bulk density, and cation exchange capacity of the clay fraction.

Future climate layers (2041–2060 and 2061–2080) were sourced from the BCC-CSM2-MR model under two Shared Socioeconomic Pathways: SSP2-4.5 (intermediate emissions) and SSP5-8.5 (fossil-fueled development, high emissions) within CMIP6. These periods are hereafter referred to as the 2050s and 2070s, respectively. Future climate layers (2041–2060 and 2061–2080) were sourced from the BCC-CSM2-MR model under SSP2-4.5 and SSP5-8.5 within CMIP6 (Wu et al., 2019). BCC-CSM2-MR was selected for its strong performance in simulating East Asian monsoon precipitation and temperature seasonality—key drivers of *P. lactiflora* distribution—particularly in the native range and analogous temperate zones (Xin et al., 2020). While multi-model ensembles are increasingly recommended to reduce GCM-specific uncertainty, the use of a single GCM is common in regional-to-global medicinal plant SDMs when computational constraints or regional performance validation is prioritized. Therefore, prioritizing this regionally validated, high-performing single GCM provides more reliable projections at the regional scale under computational constraints (Cianfrani et al., 2010; Araújo et al., 2019).

Nevertheless, this choice introduces potential model-specific bias, particularly in projections of precipitation extremes or temperature variability. Future refinements could incorporate a multi-GCM ensemble (e.g., 3–5 models with high skill in temperate Asia and Europe) to quantify uncertainty and increase robustness of range-shift predictions.

All layers were clipped to a global extent (−180° to 180° longitude, −60° to 90° latitude), resampled to a common 5 arc-minute grid using bilinear interpolation, and converted to ASCII format using SDMtoolbox 2.0 for ArcGIS. Masking was applied to exclude permanent ice and water bodies.

### 2.3 Establishment of the MaxEnt model

To identify the key environmental factors shaping the global distribution of *P. lactiflora*, the MaxEnt algorithm (version 3.4.4) was employed (Phillips et al., 2025). To avoid overfitting and ensure ecologically realistic and statistically robust predictions, we first optimized the regularization multiplier (RM) and feature classes (FC) using the ENMeval R package (v2.0.0) (Muscarella et al., 2014; Bao et al., 2022). The modelling protocol was designed to maximize robustness and minimize overfitting. All 833 spatially thinned occurrence records and the environmental layers were imported into MaxEnt. ENMeval evaluated 48 parameter combinations (RM from 0.5 to 4.0 in 0.5 increments; FC combinations: linear, quadratic, hinge, product, threshold, and all subsets) using 10-fold cross-validation and the Akaike Information Criterion corrected for small sample size (AICc). The combination with the lowest AICc (RM = 1.5, FC = linear + quadratic + hinge) was selected as optimal. The optimal settings (FC = LQH, RM = 1.5) outperformed the default MaxEnt settings (FC = LQHP, RM = 1) in validation AUC, overfitting metrics, and AICc (see **Table 1** for detailed comparison). The model was configured with 10-fold cross-validation, whereby the data were randomly partitioned into a 75% training subset and a 25% test subset in each fold (Wei-Yao et al., 2019). This approach provides an objective assessment of predictive performance on independent data and reduces the risk of inflated evaluation metrics.

**Table 1:** ENMeval results comparing default and selected parameter settings.

Type	FC	RM	AUCtrain	AUCdiff.avg	OR10.avg	ORMTP.avg	ΔAICc
Selected	LQH	1.5	0.952	0.047	0.253	0.001	0.000
Default	LQHP	1	0.949	0.068	0.412	0.004	46.37

**Figure 2:**
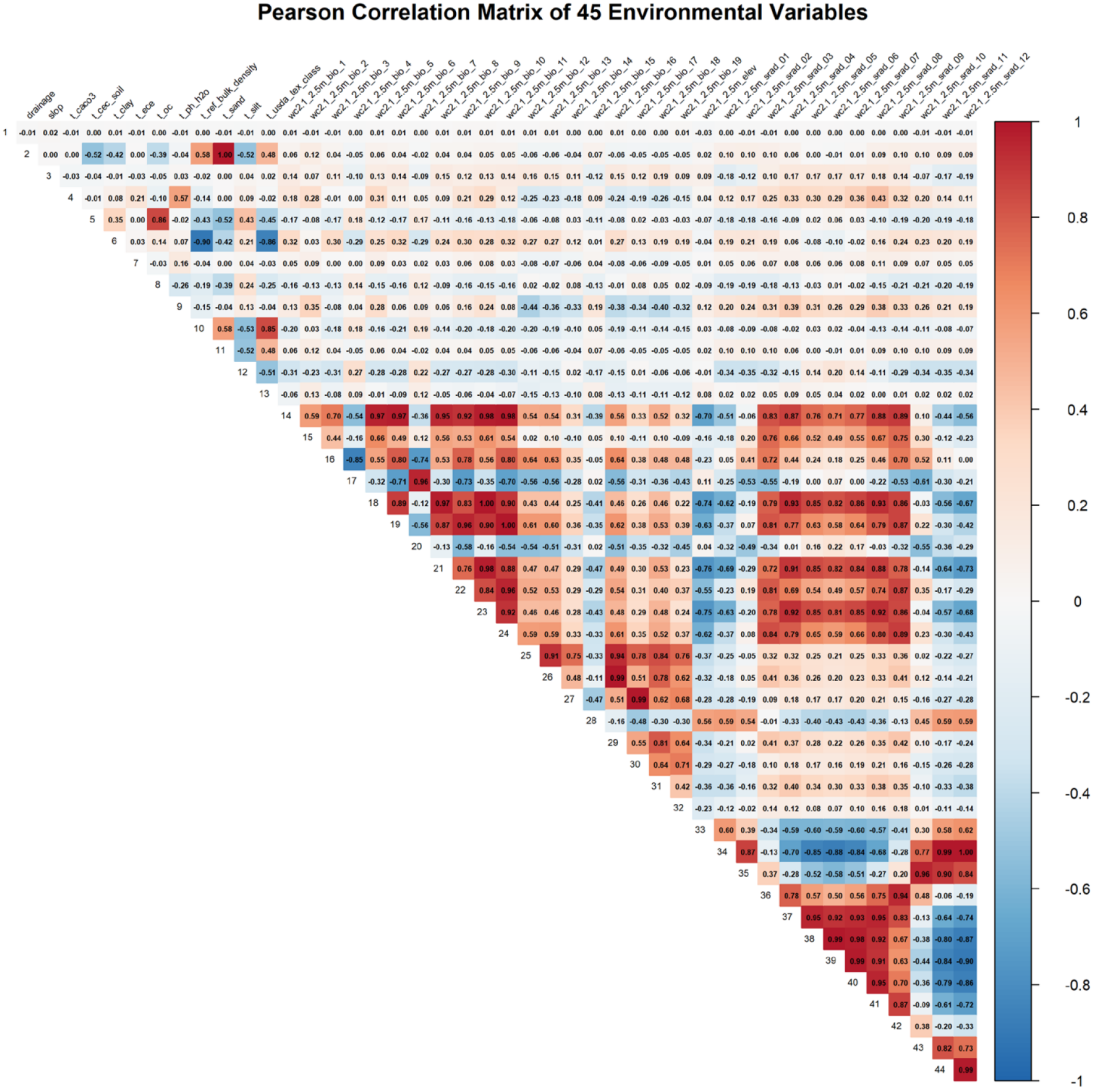
Heatmap of Pearson correlation coefficients among the initial 45 environmental variables.

To further enhance reliability, ten replicate runs were performed with different random seeds. A preliminary model using the full set of 45 environmental variables was first executed, and jackknife analysis was applied to estimate the individual contribution of each predictor (Wolter, 2007). Because strong multicollinearity was detected among the initial 45 variables, collinearity was subsequently diagnosed using ENMTools (Warren et al., 2021). Pairs of variables with a Pearson correlation coefficient |r| ≥ 0.80 were identified, and in each pair, the variable with the lower permutation importance in the preliminary model was removed. The complete Pearson correlation matrix heatmap for the initial 45 environmental predictor variables is shown in **Figure 2**. The color scale ranges from blue (−1, strong negative correlation) to red (+1, strong positive correlation), with white indicating no correlation. Absolute values ≥ 0.80 are considered highly collinear (threshold for removal) and are visually prominent in red/blue. Variables are labeled along the axes. This matrix was calculated across the entire global study area using ENMTools. This iterative screening process ultimately retained 12 non-redundant, biologically meaningful predictors.

The final MaxEnt model was then rerun using only these 12 selected variables with the optimized settings (RM = 1.5, FC = linear + quadratic + hinge) while maintaining the same 10-fold cross-validation settings, auto-feature selection, regularization multiplier (β=1.5), and 10,000 background points. The definitive percent contribution and permutation importance of each retained predictor are reported in **Table 2**.

**Table 2:** Environmental factors and their contribution rates after screening.

Variable	Description	Unit
bio_4	Temperature Seasonality (Standard Deviation * 100)	°C
bio_11	Mean Temperature of Coldest Quarter	°C
bio_15	Precipitation Seasonality (Coefficient of Variation)	mm
bio_18	Precipitation of Warmest Quarter	\
bio_19	Precipitation of Coldest Quarter	\
Elevation	altitude	m
Slope gradient	Gradient of the terrain	°
drainage	Drainage class	\
t_silt	Topsoil Silt Fraction	% wt.
t_clay	Topsoil Clay Fraction	% wt.
srad07	Solar radiation in July	KJ/m^2^/day
srad11	Solar radiation in November	KJ/m^2^/day

### 2.4 Data processing of prediction results and visual analysis

The MaxEnt logistic output (suitability values ranging from 0 to 1) was reclassified using a consistent threshold for binary and quantitative analyses. For presence/absence mapping, range-change detection, area statistics of suitable habitat, and centroid shift calculations, we applied the threshold that maximized training sensitivity plus specificity (MaxSSS; mean = 0.312 ± 0.017 across 10-fold cross-validation). This threshold is widely recommended for presence-only data because it balances omission and commission errors without requiring true absence information (Liu et al., 2005; Jiménez-Valverde and Lobo, 2007). Pixels exceeding 0.312 were classified as suitable.

To visualize relative suitability gradients and facilitate ecological interpretation, we additionally applied the Jenks natural breaks algorithm in ArcGIS to divide the continuous suitability values into four classes: unsuitable, marginally suitable, moderately suitable, and highly suitable (Chen et al., 2013). Current and future suitability layers were overlaid using the raster overlay tool to quantify spatial patterns of habitat expansion, contraction, and stability under each emission scenario–time-period combination. Shifts in the centroid of suitable habitat (>0.312) between present and future projections were calculated with SDMtoolbox 2.0.

**Figure 3:**
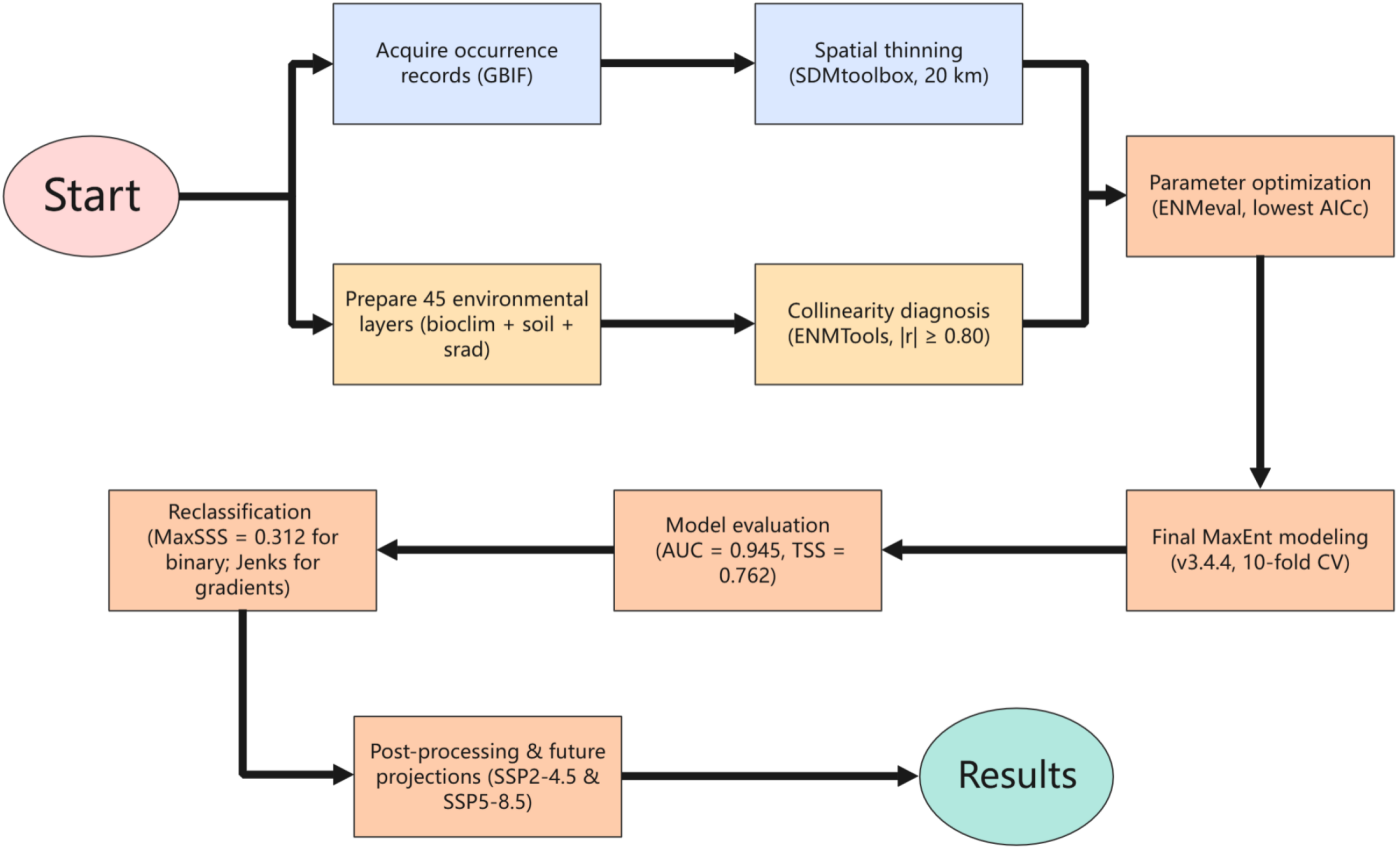
Optimized methodological workflow of the MaxEnt species distribution modeling process for *P. lactiflora*.

To enhance clarity and reproducibility, the overall methodological workflow is presented in **Figure 3**. This diagram details the sequential process from GBIF occurrence data collection and spatial thinning, environmental variable preparation and collinearity filtering, parameter tuning using ENMeval, final MaxEnt modeling and evaluation, suitability reclassification (using MaxSSS for binary analysis and Jenks for gradient visualization), to post-processing analyses including area quantification, centroid shift calculation, and projections under SSP scenarios.

## 3 Results

### 3.1 Accuracy of MaxEnt model analysis

Model performance was evaluated using two widely accepted metrics: area under the receiver operating characteristic curve (AUC) and the True Skill Statistic (TSS) (Allouche et al., 2006; Chen et al., 2013; Carrington et al., 2023; Yoon and Lee, 2023).

**Figure 4:**
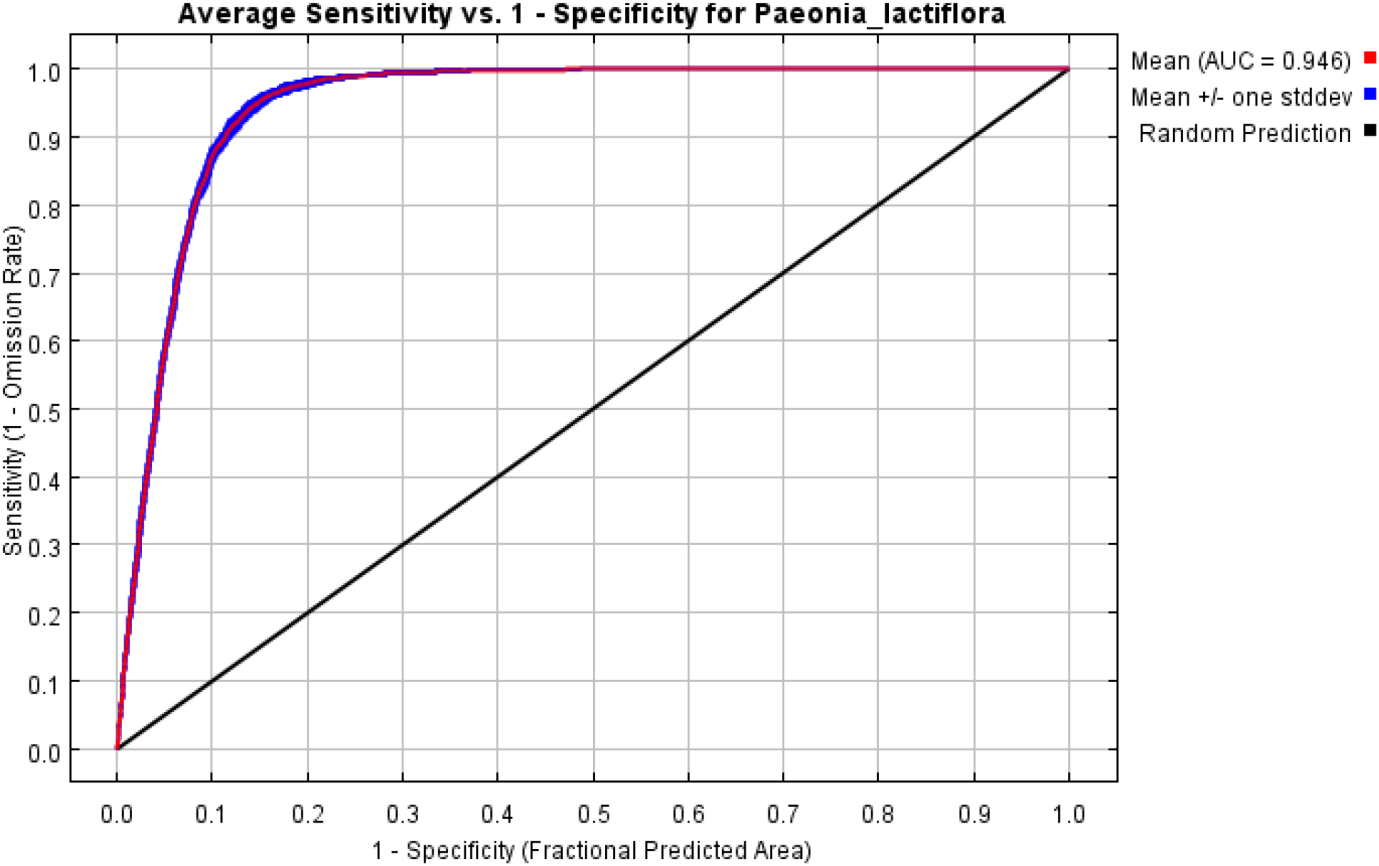
The ROC curves of the MaxEnt model for *P. lactiflora*.

The AUC measures the model’s ability to discriminate presence records from background points, independent of any specific threshold. Values >0.8 indicate good performance, whereas values >0.9 are considered excellent. In the present study, the mean test AUC across the 10 cross-validation folds was 0.945 ± 0.001 (SD), demonstrating outstanding discriminatory power (**Figure 4**; **Table 3**).

**Table 3:** AUC value and TSS value of each component module in the study.

Type	current	SSP245-2050	SSP245-2070	SSP585-2050	SSP585-2070
AUC	0.946	0.945	0.946	0.945	0.944
TSS	0.782	0.735	0.769	0.764	0.762

The TSS complements AUC by incorporating a specific threshold, thereby directly assessing classification accuracy while remaining independent of prevalence. It ranges from −1 to +1, with values >0.7 generally regarded as evidence of high predictive reliability suitable for conservation and management applications (Bradie and Leung, 2017). The mean TSS obtained here was 0.762 ± 0.018, calculated in R v4.3.2 using the package ‘dismo’.

Collectively, the high AUC and TSS values confirm that the final MaxEnt model is both highly accurate and robust, making it well-suited for mapping the current and future global distribution of suitable habitat for *P. lactiflora*.

### 3.2 Assessment of primary environmental determinants governing the geographic distribution of *P. lactiflora*

**Figure 5:**
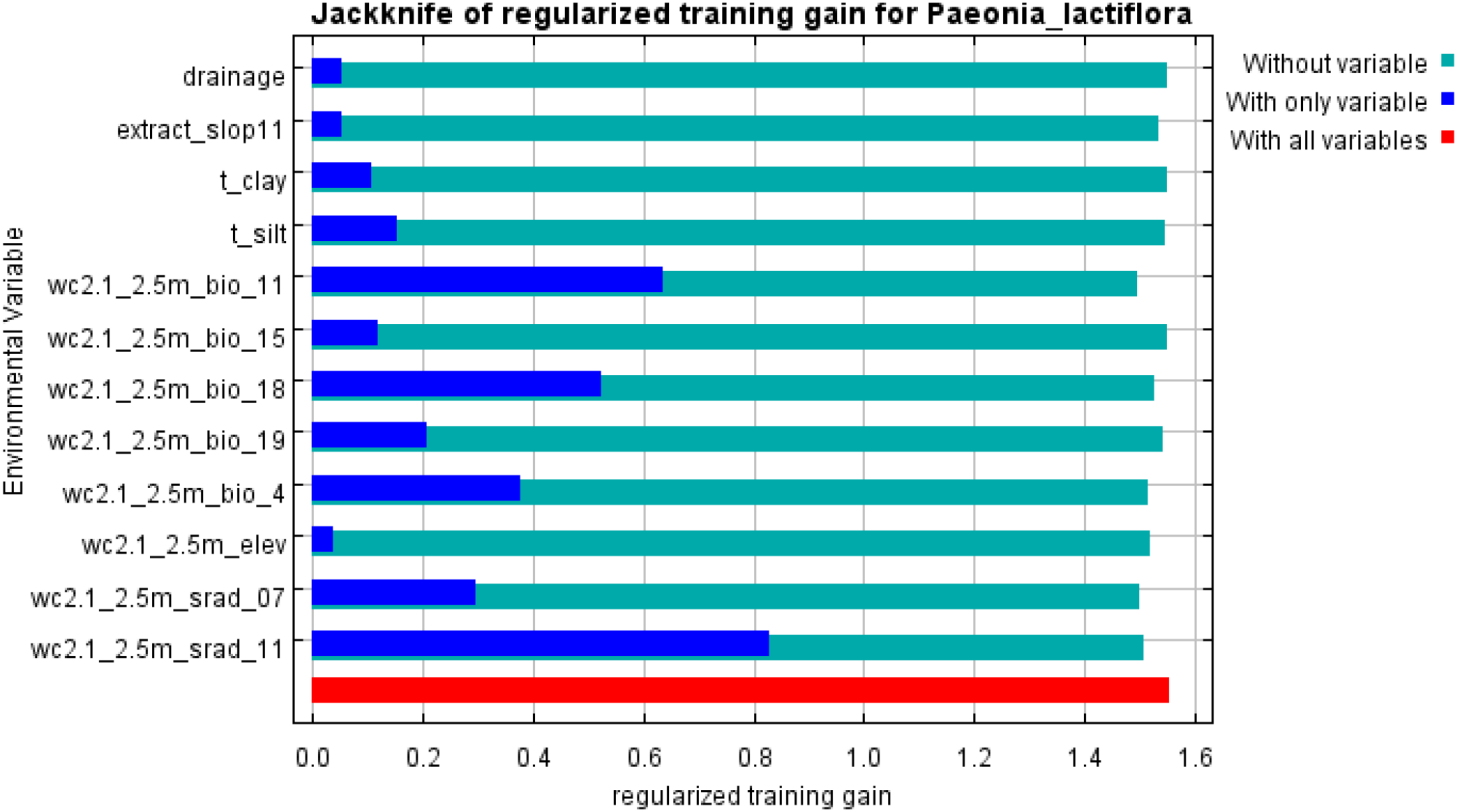
Jackknife test of environmental variables for *P. lactiflora*.

**Figure 6:**
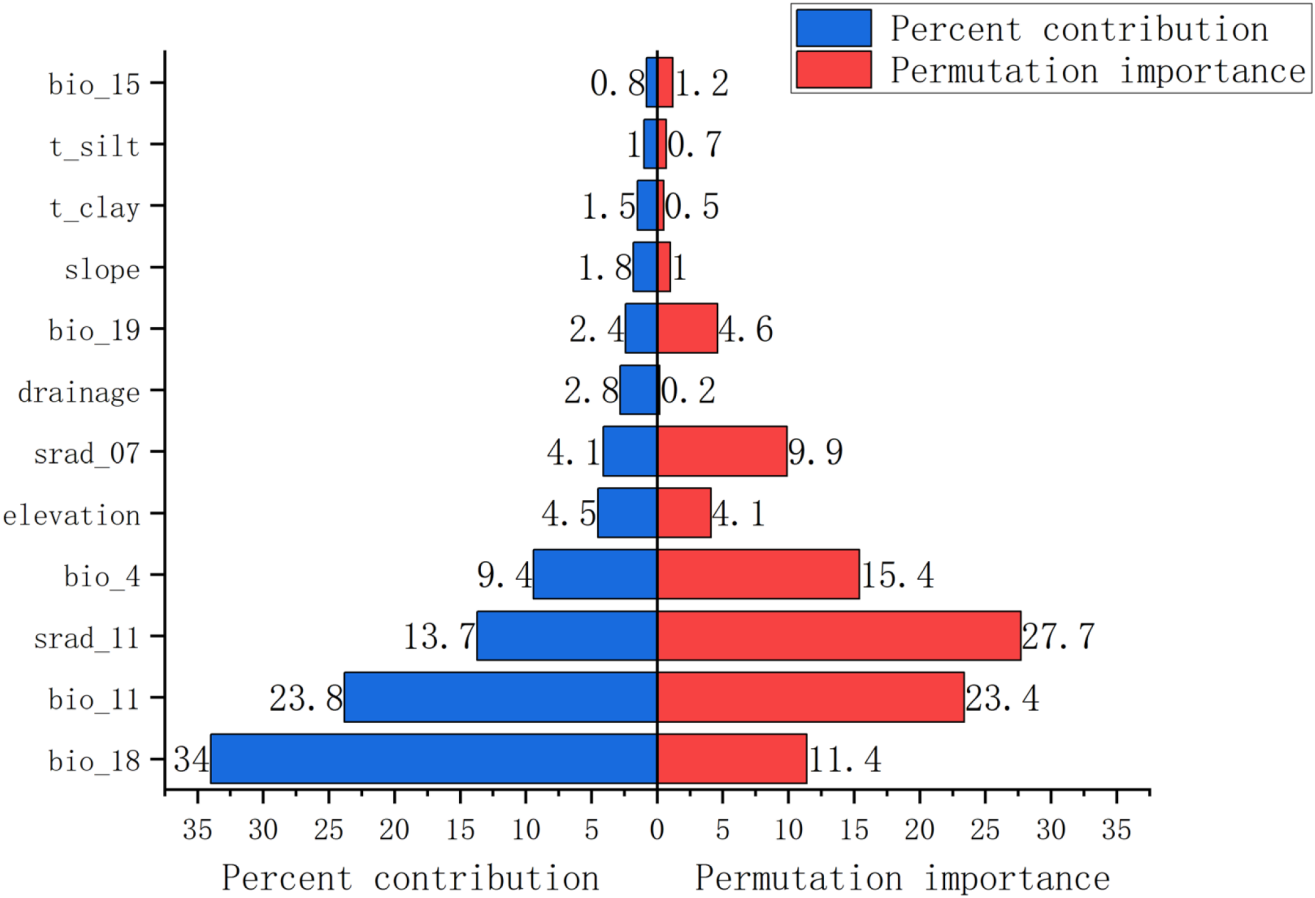
The permutation importance and percent contribution.

The relative influence of each environmental predictor was assessed using MaxEnt’s built-in jackknife procedure on regularized training gain and test AUC (McIntosh, 2016). The four dominant variables collectively accounted for the majority of model explanatory power (**Figure 5**). Precipitation of the warmest quarter (bio18), mean temperature of the coldest quarter (bio11), solar radiation in November (srad11), and temperature seasonality (bio4) emerged as the most important drivers of global habitat suitability for *P. lactiflora* (**Figure 6**).

Percent contribution reflects the increase in regularized gain attributed to each variable during model fitting, whereas permutation importance measures the drop in test AUC when the values of that variable are randomly shuffled on withheld data; the latter is generally regarded as a more reliable indicator of true predictive relevance. In the final model, bio18 exhibited the highest permutation importance, followed by bio11, bio4, and srad11 (**Figure 6**, **Table 2**).

**Figure 7:**
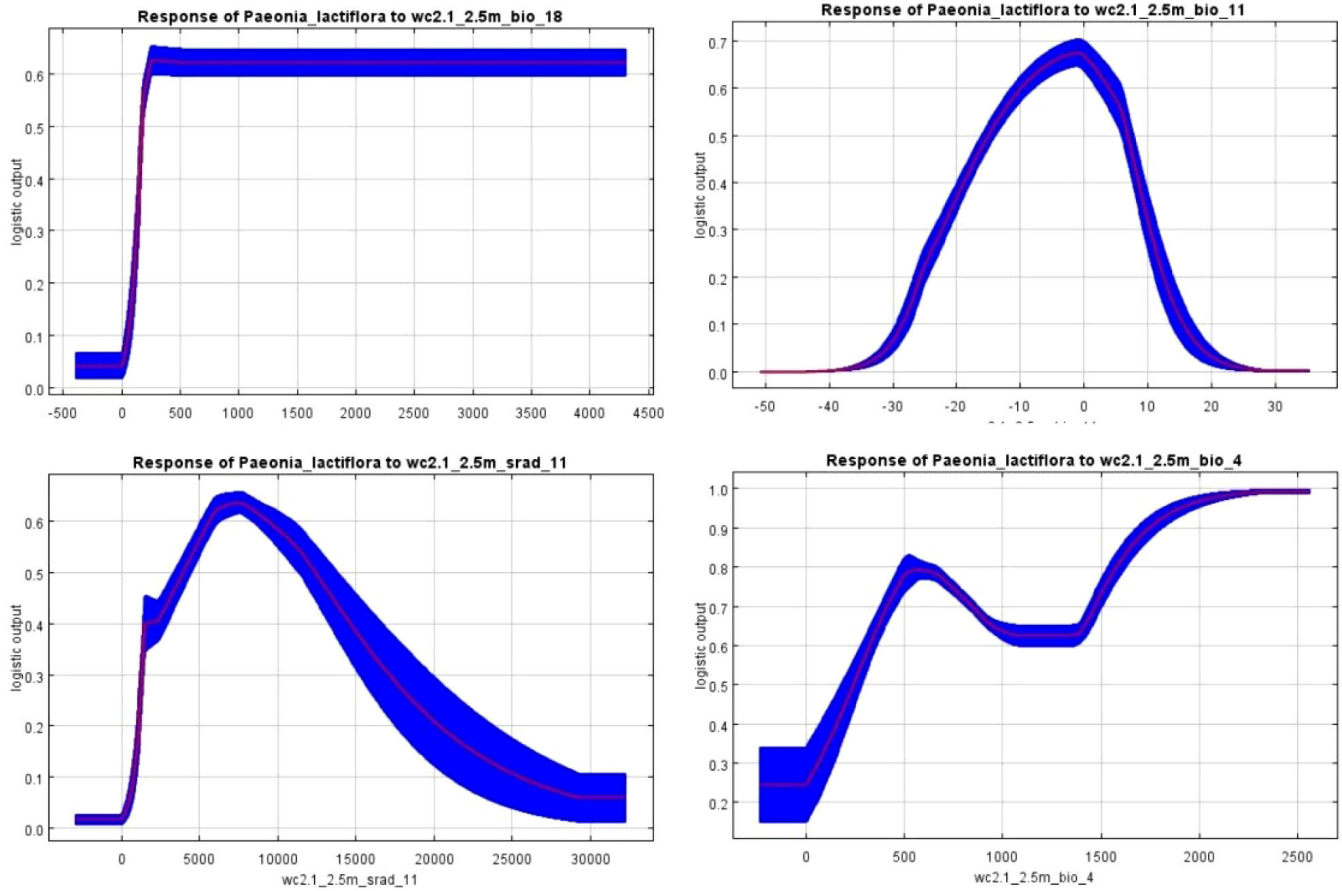
Response curves for bio18, bio11, srad11 and bio4.

Marginal response curves for these four key predictors are shown in **Figure 7**. These curves illustrate how predicted suitability changes as each environmental variable is varied while all others are held at their average sample value, thereby revealing the realized niche boundaries of the species along each gradient.

The response curves reflect the physiological tolerance and ecological niche preferences of a species. According to Shelford’s Law of Tolerance, each species possesses an ecological amplitude—that is, a tolerance range for every environmental factor, including a minimum threshold, an optimal range, and a maximum limit (Lynch and Gabriel, 1987). The response curve serves as a visual representation of this principle within the framework of mathematical modeling.

Precipitation of the warmest quarter (bio18), which represents water availability during the main growing season, was the most influential predictor. Suitability increased sharply when bio18 exceeded approximately 200 mm and reached its highest values above 280–350 mm, reflecting the species’ strong association with summer-monsoon climates and its sensitivity to growing-season drought.

Mean temperature of the coldest quarter (bio11) exerted a clear constraining effect on the northern and altitudinal range limits. Predicted suitability was highest when bio11 fell between −15 °C and 7 °C, with a pronounced optimum around −10 °C to 2 °C. Values below −15 °C or consistently above 7 °C resulted in a rapid decline in suitability, confirming the critical role of moderate winter cold for dormancy and spring regrowth.

Temperature seasonality (bio4) further restricted the species to regions with moderate annual thermal variation. Suitability declined markedly when bio4 exceeded approximately 850 (standard deviation × 100), effectively excluding continental interiors characterized by extreme temperature fluctuations.

November solar radiation (srad11) also emerged as an important driver. Optimal conditions occurred between roughly 4,000 and 12,500 kJ m⁻² day⁻¹, with suitability decreasing at both lower values (typical of higher latitudes in late autumn) and higher values (low-latitude regions).

Secondary contributions were made by elevation, soil drainage class, and precipitation of the coldest quarter, which primarily refined local-scale habitat quality within the broader climatic envelope defined by the four dominant variables.

### 3.3 Potential geographical distribution and habitat evaluation

#### 3.3.1 Spatial pattern of the current potential distribution of *P. lactiflora*

**Figure 8:**
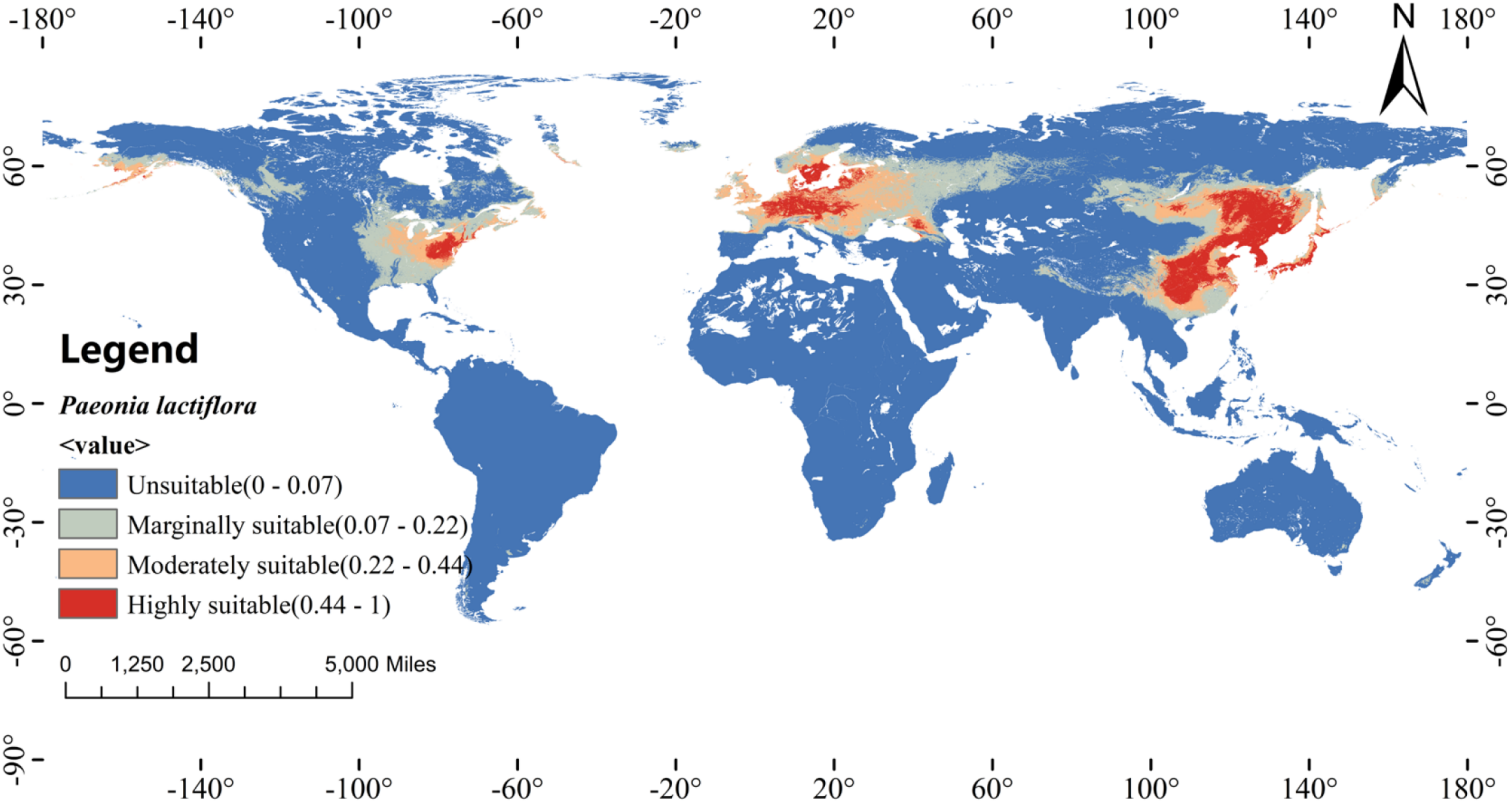
Current potential distribution of *P. lactiflora*.

Under present-day climate conditions (1970–2000), the MaxEnt model predicts the potential suitable distribution areas of peony globally, as shown in **Figure 8**. This extensive suitable range spans three continents and reflects both the species’ native East Asian distribution and the climatic analogues that support its successful cultivation and naturalization elsewhere.

For descriptive purposes, suitability classes were derived using the Jenks natural breaks classification. Highly suitable habitat (logistic value >0.44) covers 5.53 million km² and forms three distinct core areas: the traditional Chinese cultivation heartland and native range in eastern China (especially Shandong, Henan, Jiangsu, Anhui, and surrounding provinces), together with the Korean Peninsula and central Honshu, Japan; a broad belt across central Europe, centered on Germany, Poland, the Czech Republic, Austria, and adjacent lowlands; and a smaller but well-defined region in the northeastern and midwestern United States, where the species has long been cultivated and occasionally naturalized.

Moderately suitable habitat (0.22-0.44), totaling 7.06 million km², surrounds these high-suitability cores and expands continuously into neighboring temperate zones. In China, it extends northward into Northeast China, westward into parts of North and Southwest China, and southward along lower-elevation corridors. In Europe, it includes most of France, the United Kingdom, Belgium, the Netherlands, and southern Scandinavia. In North America, the band covers much of the Upper Midwest and parts of the Mid-Atlantic states.

Marginally suitable habitat (0.07-0.22) accounts for the remaining 13.72 million km² and occupies transitional climatic zones at the outer edges of the species’ realized niche. These areas include the semi-arid northwest of China and the fringes of the Qinghai–Tibet Plateau, the Mediterranean littoral (northern Italy, coastal Balkans, Greece, and western Turkey), as well as the northern Pacific coast of the United States and interior valleys of the western Cordillera. Although these regions currently support only limited cultivation or occasional escape, they share climatic features (particularly adequate warm-season precipitation and moderate winter cold) that place them within the broader ecological amplitude of the species.

#### 3.3.2 Visualization analysis of potential habitat distribution prediction for *P. lactiflora* under future climate conditions

**Figure 9:**
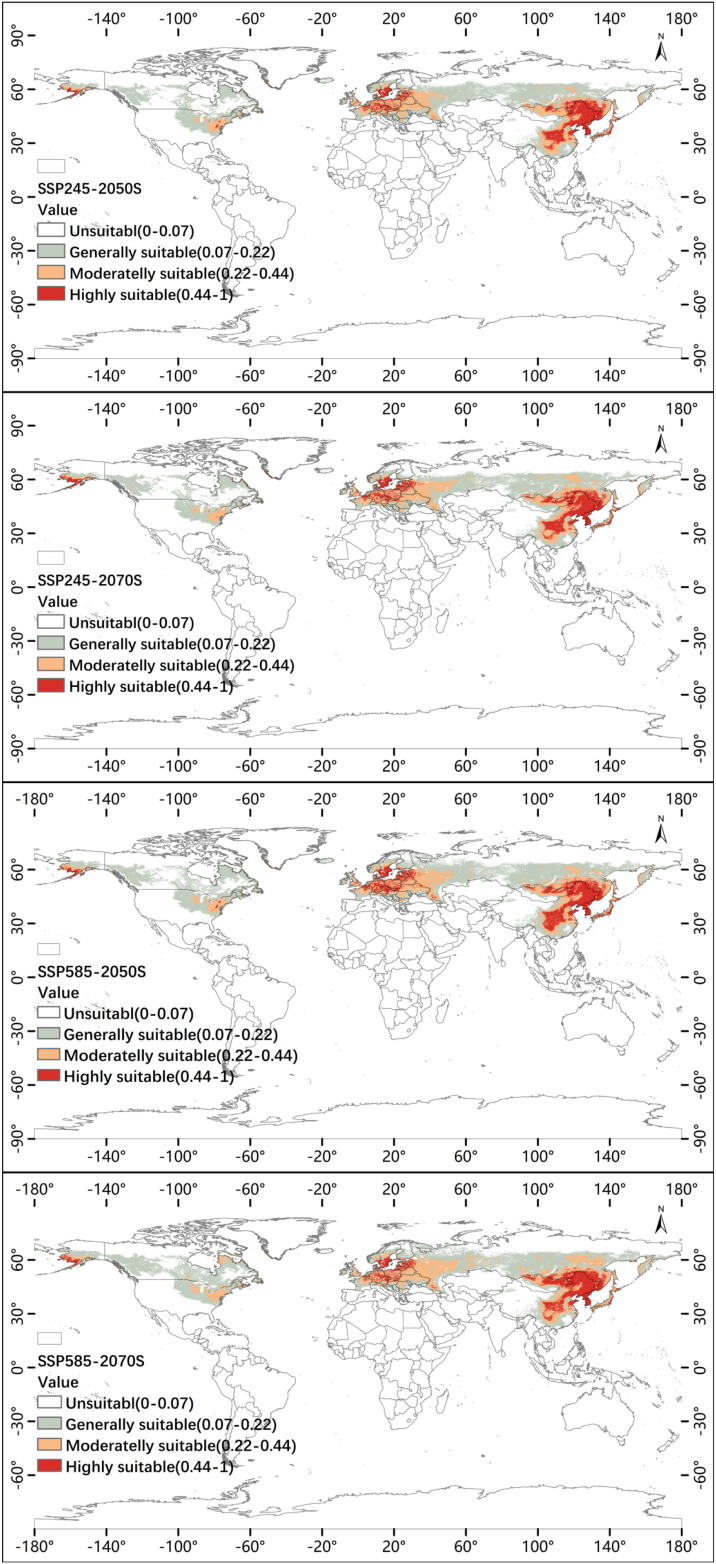
Future climate–driven potential distribution of *P. lactiflora*.

Under the two Shared Socioeconomic Pathways examined (SSP2-4.5 and SSP5-8.5) and the two future time periods (2041–2060 and 2061–2080), the MaxEnt projections consistently indicate a substantial northward and poleward expansion of suitable habitat for *P. lactiflora* (**Figure 9**).

**Figure 10:**
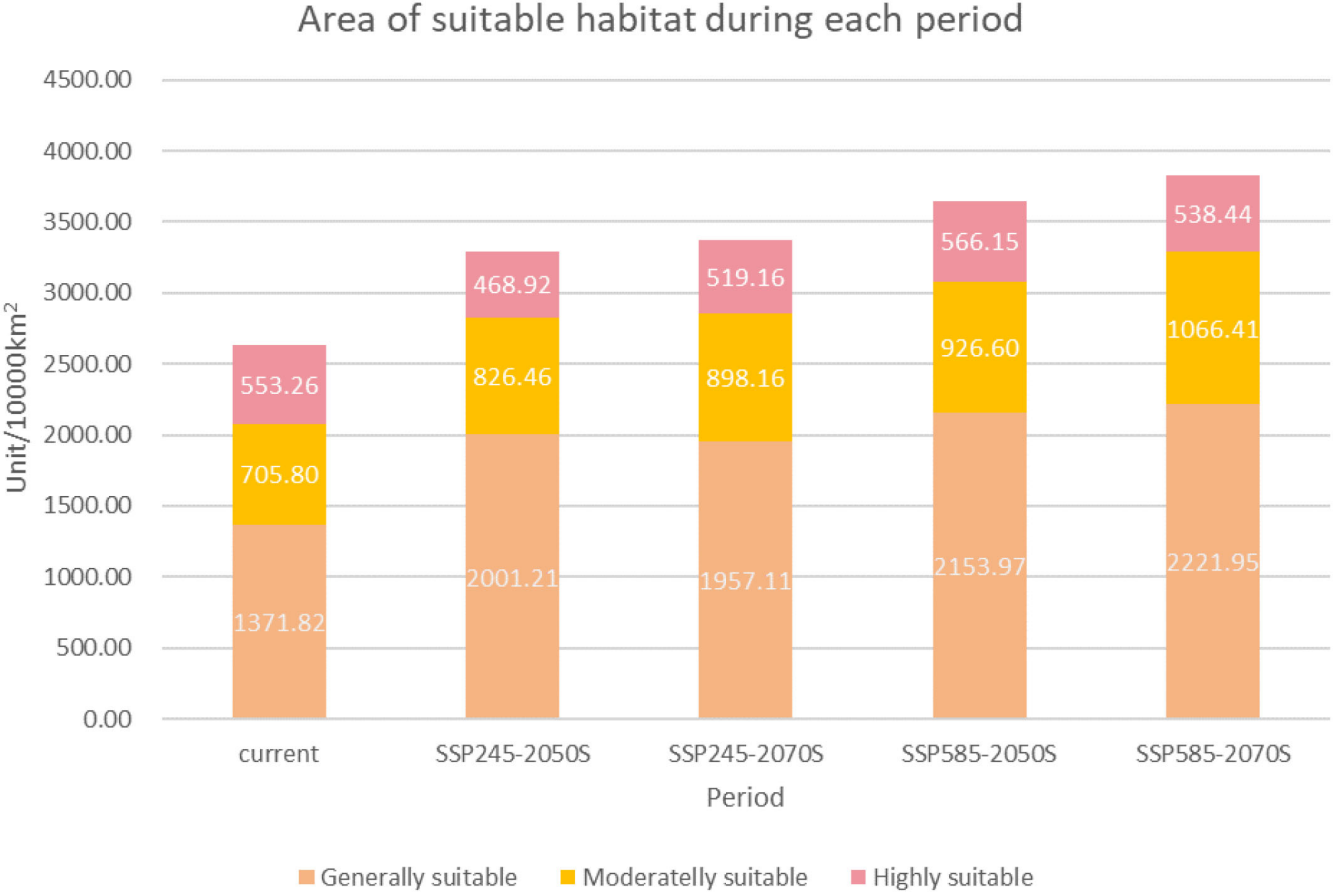
Changes in the area of different suitability classes for *P. lactiflora* under current and future climate scenarios.

For visualization of future habitat change trends, the Jenks natural breaks classification was applied. Compared with the current total suitable area of 26.31 million km², all future scenarios exhibit marked increases (**Figure 10**). In the 2041–2060 period, the total suitable area rises to 32.97 million km² under SSP2-4.5 and 33.74 million km² under SSP5-8.5. By 2061–2080, the extent continues to grow, reaching 33.74 million km² under SSP2-4.5 and 38.27 million km² under SSP5-8.5 — representing a maximum increase of nearly 45% relative to present-day conditions.

Highly suitable habitat shows more nuanced dynamics. It expands modestly in the mid-century (2041–2060) under both scenarios, but under the high-emission SSP5-8.5 pathway during 2061–2080 it contracts slightly by approximately 0.28 million km² compared with the 2041–2060 period, even as total suitable area continues to grow. This suggests that extremely high temperatures and altered seasonality under SSP5-8.5 begin to erode core habitat quality in some traditionally favorable regions by the late 21st century.

The newly suitable areas are predominantly located at higher latitudes: western Russia, southern Scandinavia, southeastern Canada, and interior portions of the northeastern United States become incorporated into the moderate- and high-suitability zones. Meanwhile, the original core regions in eastern China, the Korean Peninsula, Japan, and central Europe largely retain their high suitability under SSP2-4.5, whereas under late-century SSP5-8.5 some localized degradation is projected in the southernmost portions of these historic ranges.

#### 3.3.3 Changes in suitable habitat areas under different climate conditions

**Figure 11:**
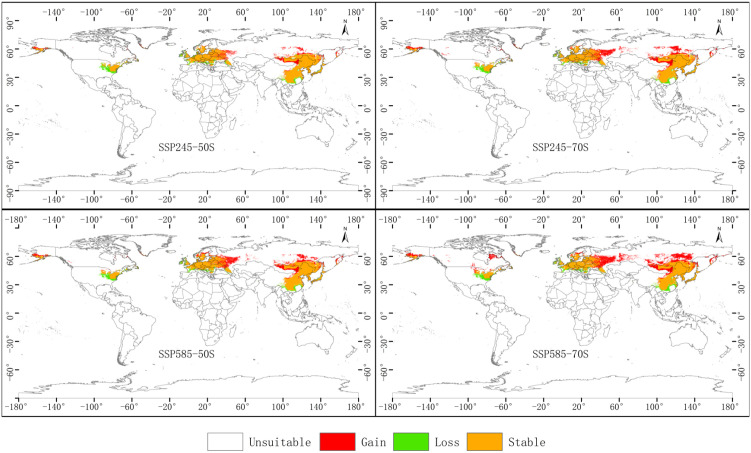
Spatial patterns of suitable habitat gain and loss for *P. lactiflora* under four future climate scenarios.

Overlay analysis in ArcGIS was used to compare current suitable habitat with projections under the four future climate scenario–period combinations. To ensure accurate quantification of habitat gain and loss, suitability was binarized using the MaxSSS threshold in the analyses presented in this section. The resulting patterns of habitat gain, loss, and stability are displayed in **Figure 11**, while the corresponding area changes are quantified in **Table 4**.

**Table 4:** Area changes of suitable habitats under four future scenarios relative to current conditions.

Status	SSP2-4.5-2050	SSP2-4.5-2070	SSP5-8.5-2050	SSP5-8.5-2070
Stable	1104.11	1113.61	1118.58	1096.60
Gain	234.79	422.02	399.68	666.83
Loss	163.02	153.40	148.49	170.20

Unit: 1×10^4^ km².

All four scenarios project a net expansion of suitable habitat, but the magnitude and spatial expression differ markedly (**Figure 9**, **Table 4**). The greatest overall increase occurs under SSP5-8.5 for 2061–2080, where newly suitable area reaches its maximum and habitat loss, although present, remains considerably smaller than gains. This results in the largest net gain among all scenarios.

In contrast, the two SSP2-4.5 scenarios (both 2041–2060 and 2061–2080) and the mid-century SSP5-8.5 scenario exhibit more moderate and balanced dynamics, with smaller areas of both gain and loss. Habitat turnover is therefore lowest under SSP2-4.5, indicating relatively stable range configuration despite overall northward expansion.

The pronounced habitat loss under late-century SSP5-8.5 primarily affects lower-latitude portions of the current range (southern China, parts of the Mediterranean fringe, and the southern United States), driven by excessive warming and altered precipitation regimes. Conversely, the most extensive gains are concentrated at the northern margins, particularly in western Russia, southern Scandinavia, and southeastern Canada, where previously unsuitable cooler regions become climatically favorable.

These results highlight that, while *P. lactiflora* is likely to benefit from net range expansion in a warming world, the highest-emission pathway in the late 21st century introduces the greatest spatial reorganization and localized degradation of historically optimal habitat.

#### 3.3.4 Spatial shifts in the centroid of suitable habitat zones

**Figure 12:**
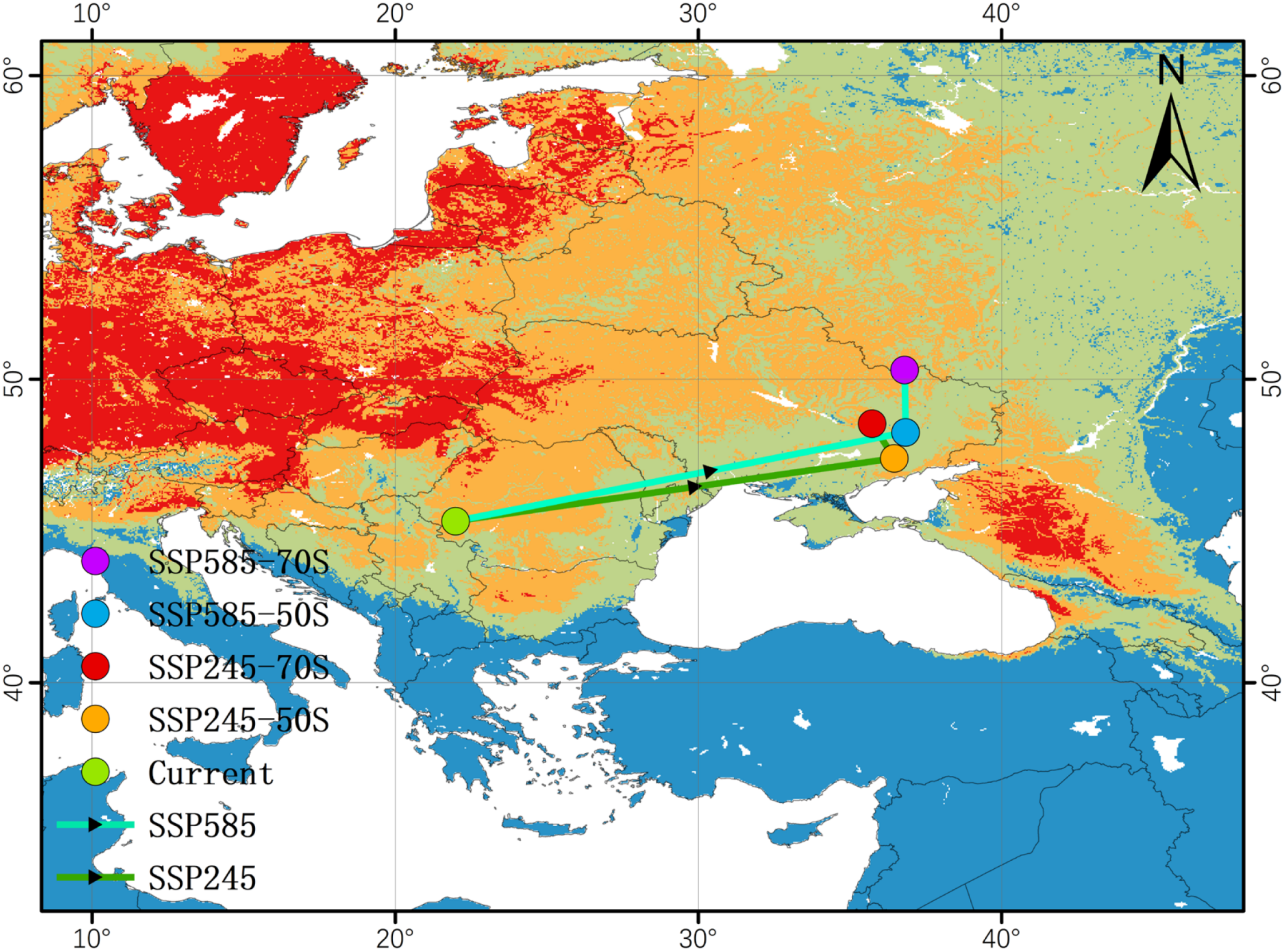
Shift map of the center of gravity in the suitable area of *P. lactiflora* under future climate scenario.

Centroid analysis provides a concise summary of the overall direction and magnitude of range displacement under climate change (Fordham et al., 2012). In this study, the current centroid of suitable habitat (>0.312) for *P. lactiflora* is located in western Romania (approximately 45.3°N, 22.0°E). Under all future scenarios, the centroid consistently shifts northeastward, crossing Moldova and reaching eastern Ukraine (**Figure 12**).

Despite the shared northeastward trajectory, the distance travelled varies markedly between emission pathways. Under the SSP5-8.5 scenarios, the centroid migrates considerably farther than under SSP2-4.5, with the longest displacement occurring in SSP5-8.5 2061–2080 (approximately 1,234.41 km from the present-day position). In contrast, centroid movement under both SSP2-4.5 time slices remains more modest (approximately 1011 km).

This pronounced difference indicates that higher-emission pathways impose stronger environmental pressure, forcing the core of the most favorable habitat to shift farther poleward and eastward in search of cooler and wetter conditions that match the species’ current climatic optimum. The shorter migration distances projected under SSP2-4.5 suggest that moderate mitigation could substantially reduce the geographic reorganization required for the persistence of high-quality habitat.

## 4 Discussion

### 4.1 Reliability and limitations of MaxEnt model prediction

The MaxEnt model developed here exhibited excellent performance (mean test AUC = 0.945 ± 0.001; mean TSS = 0.762 ± 0.018), confirming its strong discriminatory power and robustness for predicting the global distribution of *P. lactiflora*. These values compare favorably with those reported in other high-quality SDM studies of medicinal plants and temperate perennials, providing confidence that the identified environmental drivers and projected range shifts are biologically credible.

Nevertheless, several limitations inherent to the MaxEnt framework and our implementation should be acknowledged (Lissovsky et al., 2021). First, predictive accuracy remains contingent on the quality, resolution, and temporal relevance of the environmental layers. Although we used widely accepted, high-quality datasets (WorldClim 2.1, HWSD, etc.), their ~10 km resolution inevitably smooths local topographic and microclimatic variation that can be critical for a montane–lowland species such as *P. lactiflora*. Second, MaxEnt assumes that the realized niche is primarily shaped by the supplied abiotic predictors and that species–environment relationships remain relatively stable over time (Phillips and Dudík, 2008; Bradie and Leung, 2017). In reality, biotic interactions (e.g., competition with invasive species, herbivory, or mutualisms) and anthropogenic factors (e.g., land-use change, urbanization, and historical cultivation) also exert substantial influence on local presence and abundance. These processes were not explicitly incorporated and may lead to overestimation of suitability in heavily modified landscapes (particularly in Europe and eastern North America) or underestimation in regions where human management has historically facilitated persistence outside the strict climatic niche (Wisz et al., 2013; Gwitira et al., 2014).

Despite these constraints, the consistency between our modelled core areas and the known centers of traditional cultivation and naturalization lends strong support to the overall reliability of the projections. Future refinements could usefully integrate dynamic land-use layers, dispersal constraints, or ensemble approaches across multiple algorithms to further reduce uncertainty.

### 4.2 Ecological impacts of key environmental variables on *P. lactiflora* distribution

Jackknife analysis and permutation importance identified four variables that collectively dominate the realized niche of *P. lactiflora*: precipitation of the warmest quarter (bio18), mean temperature of the coldest quarter (bio11), temperature seasonality (bio4), and solar radiation in November (srad11). The overriding influence of these predictors highlights the species’ specialization for a temperate monsoon climate characterized by abundant summer rainfall, moderate winter cold, limited annual thermal amplitude, and adequate late-autumn irradiance.

The paramount importance of bio18 underscores the critical dependence of *P. lactiflora* on high moisture availability during the main growing and flowering season. This requirement aligns closely with the summer-monsoon precipitation regime of its native East Asian range and explains both its absence from Mediterranean winter-rainfall regions and its successful cultivation in similarly summer-wet areas of central Europe and eastern North America (Chetvertak et al., 2025).

The strong constraining effect of bio11 reflects the necessity of a pronounced but not extreme cold period for proper dormancy induction and subsequent spring regrowth, consistent with the species’ well-documented chilling requirement and frost tolerance (Pescador et al., 2018; Marković et al., 2022). Similarly, the negative response to high temperature seasonality (bio4) indicates low tolerance for continental climates with large annual thermal fluctuations, restricting the species to oceanic or sub-oceanic temperate zones (Menzel and Sparks, 2006).

November solar radiation (srad11) emerged as an unexpectedly influential photoperiod- and energy-related cue. High suitability within a relatively narrow late-autumn irradiance window (≈4,000–12,500 kJ m⁻² day⁻¹) suggests that sufficient light penetration before leaf senescence is essential for carbohydrate translocation to roots, thereby supporting overwinter survival and the following year’s flowering (Zhao et al., 2015).

These findings substantially deepen our understanding of the ecological amplitude of *P. lactiflora* and provide physiologically interpretable thresholds that can guide both cultivation practices and conservation planning under climate change. Nonetheless, the model necessarily simplifies reality; factors not explicitly included—such as soil microbial communities, atmospheric pollutants, or fine-scale edaphic heterogeneity—may further modulate local realized niches and warrant investigation in future studies.

This study demonstrates that integrating soil properties and solar radiation variables into MaxEnt significantly enhances explanatory power for temperate herbaceous perennials, particularly by capturing fine-scale edaphic constraints and photoperiodic cues often overlooked in purely climatic SDMs. The emergence of November solar radiation as a dominant driver highlights the potential of radiation data to refine niche predictions in mid-latitude species, where autumn light availability critically influences resource storage and overwintering success. These findings suggest that future SDM applications for medicinal plants and other temperate herbs should routinely incorporate solar radiation and soil layers to improve mechanistic understanding and predictive accuracy under climate change scenarios.

### 4.3 Global-scale ecological mechanisms underlying distribution patterns

The distribution of *P. lactiflora* at a global scale reflects a suite of interacting ecological mechanisms that operate across continental climate gradients, biogeographic barriers, and species-specific traits. As a temperate herbaceous perennial, *P. lactiflora* exemplifies how global climate patterns shape plant niches through interactions between abiotic drivers and biotic processes.

Precipitation during the warmest quarter (bio18) acts as the primary limiter, reflecting the species’ dependence on summer monsoon regimes typical of East Asia. This mechanism aligns with broader global patterns where herbaceous perennials in humid temperate zones rely on seasonal moisture pulses for rapid growth and reproduction, distinguishing them from woody species that tolerate greater drought through deeper roots (Wang et al., 2020; Xing et al., 2024). At continental scales, this creates disjunct distributions, with climatic analogues in central Europe and North America enabling naturalization, but biogeographic barriers (e.g., oceans, deserts) prevent natural dispersal from the native range, underscoring human-mediated introduction as a key mechanism for global expansion.

Winter temperature (bio11) and seasonality (bio4) further constrain poleward limits, enforcing a chilling requirement for dormancy break—a common mechanism in temperate perennials to synchronize growth with seasonal cycles and avoid frost damage (Lubbe et al., 2021). Globally, this interacts with latitudinal gradients, where increasing temperature variability in continental interiors excludes the species, favoring oceanic climates with buffered thermal regimes (Gillespie and Volaire, 2017). November solar radiation (srad11) introduces a photoperiodic dimension, likely regulating autumn resource allocation to roots, enhancing overwintering success in mid-latitude zones but limiting equatorward expansion where daylength cues are less pronounced (Petterle et al., 2013).

These abiotic mechanisms are modulated by biotic interactions at global scales, such as competition with invasive species in newly suitable areas or facilitation by soil microbes in authentic production regions—factors our model indirectly captures through edaphic variables but may underestimate in projections (Wisz et al., 2013). Climate change amplifies these dynamics, as seen in projected northeastward shifts driven by poleward migration of isothermal lines—a mechanism observed in many herbaceous taxa (Chen et al., 2011). However, dispersal limitations and habitat fragmentation could hinder realization of these projections, potentially leading to extinction debt in trailing edges (Tilman et al., 1994). Overall, P. lactiflora’s global patterns underscore how climate envelopes interact with evolutionary history and anthropogenic factors, offering a model for predicting medicinal plant vulnerabilities in a changing world.

### 4.4 Trends in the distribution of *P. lactiflora* under future climate scenarios

Our projections reveal a consistent expansion of suitable habitat for *P. lactiflora* under all examined scenarios, accompanied by a clear northeastward shift of the centroid of highly suitable areas. These twin patterns—range enlargement coupled with poleward and eastward displacement—are typical of many temperate East Asian perennials responding to warming and changing precipitation regimes.

The magnitude of both expansion and centroid shift is markedly greater under SSP5-8.5 than SSP2-4.5, particularly by 2061–2080. The longer migration distances projected under the high-emission pathway reflect stronger climatic pressure, forcing the core of optimal habitat to track cooler and wetter conditions farther into higher-latitude regions of eastern Europe and western Russia.

This climate-driven reorganization has dual implications for conservation and sustainable utilization. On one hand, the substantial northward extension creates new opportunities for cultivation in areas that are currently too cool, potentially broadening the resource base for Paeoniae Radix. On the other hand, localized loss of highly suitable habitat at the southern and western margins of the current range—especially under late-century SSP5-8.5—risks reducing population stability and genetic diversity in traditional production regions, while increasing competitive interactions with resident species in newly colonized northern zones.

These findings underscore the urgent need for proactive, forward-looking conservation strategies. Priority actions should include: (i) establishing monitored *ex-situ* collections from across the current native and cultivated range, (ii) identifying and protecting future climatic refugia identified in this study, and (iii) developing assisted migration or new cultivation guidelines for emerging high-suitability areas. Timely implementation of such measures will be essential to safeguard both wild germplasm and the long-term security of this economically and culturally important medicinal resource in a warming world.

### 4.5 Strategic suggestions for the sustainable use of *P. lactiflora* germplasm resources

The findings of this study provide clear, actionable guidance for the long-term conservation and sustainable utilization of *P. lactiflora* germplasm in a changing climate.

Firstly, in regions currently identified as highly suitable—particularly eastern China, the Korean Peninsula, central Europe, and the northeastern United States—conservation measures should be significantly strengthened. Establishing or expanding nature reserves, implementing stricter land-use regulations, and reducing habitat fragmentation will help protect genetically diverse wild and cultivated populations that have co-evolved with local conditions over centuries.

Secondly, for areas projected to become highly suitable in the future (especially western Russia, southeastern Canada, and southern Scandinavia under SSP5-8.5), proactive planning and ecological restoration are recommended. Creating connectivity corridors, improving soil conditions where needed, and conducting pilot assisted-migration programs using provenances from trailing-edge populations can facilitate successful range expansion and prevent future genetic bottlenecks.

Lastly, given the consistent northeastward shift of the suitability centroid, long-term dynamic monitoring networks should be established across the entire current and future range. At the same time, international cooperation among East Asia, Europe, and North America must be enhanced through shared databases, joint field surveys, and coordinated research initiatives to track real-time responses and refine adaptive management strategies effectively.

Implementing these integrated, forward-looking measures will be essential to ensure both the ecological persistence and continued medicinal availability of this globally significant species throughout the 21st century.

## 5 Conclusions

This study employed an optimized MaxEnt model coupled with ArcGIS spatial analysis to assess the global habitat suitability of *P. lactiflora* under present-day and future climate conditions. The excellent model performance (mean AUC = 0.945, TSS = 0.762) confirms its reliability for large-scale ecological forecasting.

Precipitation of the warmest quarter, mean temperature of the coldest quarter, temperature seasonality, and November solar radiation emerged as the dominant constraints on the species’ realized niche, collectively explaining its restriction to temperate summer-wet climates with moderate winters and stable annual thermal regimes.

Currently, highly suitable habitat is concentrated in East Asia, central Europe, and the northeastern and midwestern United States. All future scenarios (SSP2-4.5 and SSP5-8.5, 2041–2060 and 2061–2080) project a substantial expansion of suitable area, indicating that *P. lactiflora* is likely to benefit from moderate warming in the coming decades. However, under the high-emission SSP5-8.5 pathway by late century, core habitat quality declines in parts of the current range, accompanied by pronounced northeastward displacement of the suitability centroid.

These results highlight both opportunities and risks: while new cultivation regions will open at higher latitudes, traditional production areas face increasing climatic stress. Effective long-term conservation and sustainable utilization of *P. lactiflora* germplasm will therefore require (i) reinforced protection of existing high-suitability zones, (ii) proactive preparation of emerging suitable regions, and (iii) enhanced international monitoring and collaboration. Future refinements should prioritize incorporation of dynamic land-use layers, dispersal kernels, and biotic interactions (particularly competition and herbivory) to better distinguish realizable from fundamental niche space.

## Data Availability

The original contributions presented in the study are included in the article/supplementary material. Further inquiries can be directed to the corresponding authors.

## References

[B1] Allouche O. Tsoar A. Kadmon R. (2006). Assessing the accuracy of species distribution models: prevalence, kappa and the true skill statistic (TSS). J. Appl. Ecol. 43, 1223–1232. doi: 10.1111/j.1365-2664.2006.01214.x

[B2] Araújo M. B. Anderson R. P. Barbosa A. M. Beale C. M. Dormann C. F. Early R. . (2019). Standards for distribution models in biodiversity assessments. Sci. Adv. 5, eaat4858. doi: 10.1126/sciadv.aat4858, PMID: 30746437 PMC6357756

[B3] Banavar J. R. Maritan A. Volkov I. (2010). Applications of the principle of maximum entropy: from physics to ecology. J. Physics: Condensed Matter 22, 63101. doi: 10.1088/0953-8984/22/6/063101, PMID: 21389359

[B4] Bao R. Li X. Zheng J. (2022). Feature tuning improves MaxEnt predictions of the potential distribution of *Pedicularis longiflora* and its variant. PeerJ 10, e13337. doi: 10.7717/peerj.13337, PMID: 35529480 PMC9074863

[B5] Bi Y. Q. Zhang M. X. Chen Y. Wang A. X. Li M. H. (2022). Applying Biomod2 for modeling species suitable habitats: a case study of *Paeonia lactiflora* in China. Zhongguo Zhong Yao Za Zhi 47, 376–384. doi: 10.19540/j.cnki.cjcmm.20211023.101, PMID: 35178979

[B6] Bradie J. Leung B. (2017). A quantitative synthesis of the importance of variables used in MaxEnt species distribution models. J. Biogeography 44, 1344–1361. doi: 10.1111/jbi.12894

[B7] Carrington A. M. Manuel D. G. Fieguth P. W. Ramsay T. Osmani V. Wernly B. . (2023). Deep ROC analysis and AUC as balanced average accuracy for improved classifier selection, audit, and explanation. IEEE Trans. Pattern Anal. Mach. Intell. 45, 329–341. doi: 10.1109/TPAMI.2022.3145392, PMID: 35077357

[B8] Chen I.-C. Hill J. K. Ohlemüller R. Roy D. B. Thomas C. D. (2011). Rapid range shifts of species associated with high levels of climate warming. Science 333, 1024–1026. doi: 10.1126/science.1206432, PMID: 21852500

[B9] Chen J. Yang S. Li H. Zhang B. Lv J. (2013). Research on geographical environment unit division based on the method of natural breaks (Jenks). Int. Arch. Photogrammetry Remote Sens. Spatial Inf. Sci. 40, 47–50. doi: 10.5194/isprsarchives-XL-4-W3-47-2013

[B10] Chetvertak T. Diuzhykova T. Hryshko S. Nepsha O. Tutova H. (2025). The precipitation levels during the warmest quarter are the primary factor influencing the spatial distribution of *Opatrum sabulosum*. Biosyst. Diversity 33, e2507–e2507. doi: 10.15421/012507

[B11] Cianfrani C. Le Lay G. Hirzel A. H. Loy A. (2010). Do habitat suitability models reliably predict the recovery areas of threatened species? J. Appl. Ecol. 47, 421–430. doi: 10.1111/j.1365-2664.2010.01781.x

[B12] Elith J. Phillips S. J. Hastie T. Dudík M. Chee Y. E. Yates C. J. (2011). A statistical explanation of MaxEnt for ecologists. Diversity Distributions 17, 43–57. doi: 10.1111/j.1472-4642.2010.00725.x

[B13] Fick S. E. Hijmans R. J. (2017). WorldClim 2: new 1-km spatial resolution climate surfaces for global land areas. Int. J. Climatology 37, 4302–4315. doi: 10.1002/joc.5086

[B14] Fordham D. A. Akçakaya H. R. Araújo M. B. Elith J. Keith D. A. Pearson R. . (2012). Plant extinction risk under climate change: are forecast range shifts alone a good indicator of species vulnerability to global warming? Global Change Biol. 18, 1357–1371. doi: 10.1111/j.1365-2486.2011.02614.x

[B15] Gillespie L. M. Volaire F. A. (2017). Are winter and summer dormancy symmetrical seasonal adaptive strategies? The case of temperate herbaceous perennials. Ann. Bot. 119, 311–323. doi: 10.1093/aob/mcw264, PMID: 28087658 PMC5314652

[B16] Gwitira I. Murwira A. Shekede M. D. Masocha M. Chapano C. (2014). Precipitation of the warmest quarter and temperature of the warmest month are key to understanding the effect of climate change on plant species diversity in southern African savannah. Afr. J. Ecol. 52, 209–216. doi: 10.1111/aje.12105

[B17] Halvorsen R. Mazzoni S. Dirksen J. W. Næsset E. Gobakken T. Ohlson M. (2016). How important are choice of model selection method and spatial autocorrelation of presence data for distribution modelling by MaxEnt? Ecol. Model. 328, 108–118. doi: 10.1016/j.ecolmodel.2016.02.021

[B18] He D.-Y. Dai S.-M. (2011). Anti-inflammatory and immunomodulatory effects of *Paeonia lactiflora* Pall., a traditional Chinese herbal medicine. Front. Pharmacol. 2. doi: 10.3389/fphar.2011.00010, PMID: 21687505 PMC3108611

[B19] Huang H. (2011). Plant diversity and conservation in China: planning a strategic bioresource for a sustainable future. Botanical J. Linn. Soc. 166, 282–300. doi: 10.1111/j.1095-8339.2011.01157.x, PMID: 22059249

[B20] Huang J. Li Y. Fu C. Chen F. Fu Q. Dai A. . (2017). Dryland climate change: recent progress and challenges. Rev. Geophysics 55, 719–778. doi: 10.1002/2016RG000550

[B21] Isaac N. J. Jarzyna M. A. Keil P. Dambly L. I. Boersch-Supan P. H. Browning E. . (2020). Data integration for large-scale models of species distributions. Trends Ecol. Evol. 35, 56–67. doi: 10.1016/j.tree.2019.08.006, PMID: 31676190

[B22] Jaynes E. T. (1957). Information theory and statistical mechanics. Phys. Rev. 106, 620–630. doi: 10.1103/PhysRev.106.620

[B23] Jiménez-Valverde A. Lobo J. M. (2007). Threshold criteria for conversion of probability of species presence to either–or presence–absence. Acta Oecologica 31, 361–369. doi: 10.1016/j.actao.2007.02.001

[B24] Li Z. Liu Y. Zeng H. (2022). Application of the MaxEnt model in improving the accuracy of ecological red line identification: a case study of Zhanjiang, China. Ecol. Indic. 137, 108767. doi: 10.1016/j.ecolind.2022.108767

[B25] Lissovsky A. A. Dudov S. V. (2021). Species-distribution modeling: advantages and limitations of its application. 2. MaxEnt. Biol. Bull. Rev. 11, 265–275. doi: 10.1134/S2079086421030087

[B26] Lissovsky A. Dudov S. Obolenskaya E. (2021). Species-distribution modeling: advantages and limitations of its application. 1. General approaches. Biol. Bull. Rev. 11, 254–264. doi: 10.1134/S2079086421030075

[B27] Liu C. Berry P. M. Dawson T. P. Pearson R. G. (2005). Selecting thresholds of occurrence in the prediction of species distributions. Ecography 28, 385–393. doi: 10.1111/j.0906-7590.2005.03957.x

[B28] Lubbe F. C. Klimešová J. Henry H. A. L. (2021). Winter belowground: changing winters and the perennating organs of herbaceous plants. Funct. Ecol. 35, 1627–1639. doi: 10.1111/1365-2435.13858

[B29] Lynch M. Gabriel W. (1987). Environmental tolerance. Am. Nat. 129, 283–303. doi: 10.1086/284635

[B30] Marković T. Prijić Ž. Xue J. Zhang X. Radanović D. Ren X. . (2022). Seed traits associated with dormancy and germination of herbaceous peonies native to Serbia and China. Horticulturae 8, 585. doi: 10.3390/horticulturae8070585

[B31] McIntosh A. (2016). The jackknife estimation method. arXiv preprint. arXiv:1606.00497. doi: 10.48550/arXiv.1606.00497

[B32] Menzel A. Sparks T. (2006). “ Temperature and plant development: phenology and seasonality,” in Plant Growth and Climate Change, (Oxford: Blackwell Publishing) 70–95. doi: 10.1002/9780470988695

[B33] Meyer V. D. Köhler P. Smit N. T. Lipp J. S. Wei B. Mollenhauer G. . (2025). Dominant control of temperature on (sub-)tropical soil carbon turnover. Nat. Commun. 16, 4530. doi: 10.1038/s41467-025-59013-9, PMID: 40374595 PMC12081612

[B34] Muscarella R. Galante P. J. Soley-Guardia M. Boria R. A. Kass J. M. Uriarte M. . (2014). ENMeval: an R package for conducting spatially independent evaluations and estimating optimal model complexity for MaxEnt ecological niche models. Methods Ecol. Evol. 5, 1198–1205. doi: 10.1111/2041-210X.12261

[B35] Ni M. Vellend M. (2024). Soil properties constrain predicted poleward migration of plants under climate change. New Phytol. 241, 131–141. doi: 10.1111/nph.19164, PMID: 37525059

[B36] Pescador D. S. Sánchez A. M. Luzuriaga A. L. Sierra-Almeida A. Escudero A. (2018). Winter is coming: plant freezing resistance as a key functional trait for the assembly of annual Mediterranean communities. Ann. Bot. 121, 335–344. doi: 10.1093/aob/mcx166, PMID: 29300824 PMC5808800

[B37] Petterle A. Karlberg A. Bhalerao R. P. (2013). Daylength mediated control of seasonal growth patterns in perennial trees. Curr. Opin. Plant Biol. 16, 301–306. doi: 10.1016/j.pbi.2013.02.006, PMID: 23473967

[B38] Phillips S. J. Dudík M. (2008). Modeling of species distributions with MaxEnt: new extensions and a comprehensive evaluation. Ecography 31, 161–175. doi: 10.1111/j.0906-7590.2008.05203.x

[B39] Phillips S. J. Dudík M. Schapire R. E. (2004). A maximum entropy approach to species distribution modeling. Proc. Twenty-first Int. Conf. Mach. Learn. 83, 655–662. doi: 10.1145/1015330

[B40] Phillips S. J. Dudík M. Schapire R. E. (2025). *Maxent software for modeling* sp*ecies niches and distributions (version 3.4.4)* ( American Museum of Natural History). Available online at: http://biodiversityinformatics.amnh.org/open_source/maxent/.

[B41] Radosavljevic A. Anderson R. P. (2014). Making better MaxEnt models of species distributions: complexity, overfitting and evaluation. J. Biogeography 41, 629–643. doi: 10.1111/jbi.12227

[B42] Scott L. M. Janikas M. V. (2009). Spatial statistics in ArcGIS. In: Fischer MM and Getis A (eds) Handbook of Applied Spatial Analysis: Software Tools, Methods and Applications. Berlin: Springer, pp. 27–41. doi: 10.1007/978-3-642-03647-7_2

[B43] Shagjjava O. Dalaikhuub U. Gurbazar B.-Y. Bayasgalankhuu B. G. L. (2016). Comparison study of the physiology and simple phenolic content in cultivated and wild plants of *Paeonia lactiflora* pall. J. Anim. Plant Sci. 28, 4471–4478. Available online at: https://www.cabidigitallibrary.org/doi/full/10.5555/20163222738

[B44] Shore J. Johnson R. (1980). Axiomatic derivation of the principle of maximum entropy and the principle of minimum cross-entropy. IEEE Trans. Inf. Theory 26, 26–37. doi: 10.1109/TIT.1980.1056144

[B45] Slessarev E. W. Feng X. Bingham N. L. Chadwick O. A. (2019). Landscape age as a major control on the geography of soil weathering. Global Biogeochemical Cycles 33, 1513–1531. doi: 10.1029/2019GB006266

[B46] Tilman D. May R. M. Lehman C. L. Nowak M. A. (1994). Habitat destruction and the extinction debt. Nature 371, 65–66. doi: 10.1038/371065a0

[B47] Wang P. Huang K. Hu S. (2020). Distinct fine-root responses to precipitation changes in herbaceous and woody plants: a meta-analysis. New Phytol. 225, 1491–1499. doi: 10.1111/nph.16266, PMID: 31610024

[B48] Wang Y. Huo W. Wu K. Cao J. Zhao G. Zhang F. (2024). Prediction of the potentially suitable areas of *Paeonia lactiflora* in China based on MaxEnt and Marxan models. Front. Plant Sci. 15. doi: 10.3389/fpls.2024.1516251, PMID: 39850222 PMC11754415

[B49] Warren D. L. Matzke N. J. Cardillo M. Baumgartner J. B. Beaumont L. J. Turelli M. . (2021). ENMTools 1.0: an R package for comparative ecological biogeography. Ecography 44, 504–511. doi: 10.1111/ecog.05485

[B50] Wei-Yao K. Xin-Hai L. Hong-Fei Z. (2019). Optimizing MaxEnt model in the prediction of species distribution. Chin. J. Appl. Ecol. 30, 2116–2128. doi: 10.13287/j.1001-9332.201906.029, PMID: 31257787

[B51] Wisz M. S. Pottier J. Kissling W. D. Pellissier L. Lenoir J. Damgaard C. F. . (2013). The role of biotic interactions in shaping distributions and realised assemblages of species. Biol. Rev. 88, 15–30. doi: 10.1111/j.1469-185X.2012.00235.x, PMID: 22686347 PMC3561684

[B52] Wolter K. M. (2007). The jackknife method. Introduction to Variance Estimation, (New York, NY: Springer) 151–193. doi: 10.1007/978-0-387-35099-8

[B53] Wong D. W. S. Lee J. (2005). Statistical Analysis of Geographic Information with ArcView GIS and Arcgis. (Hoboken, NJ, USA: Wiley).

[B54] Wu T. Lu Y. Fang Y. Xin X. Li L. Li W. . (2019). The Beijing Climate Center Climate System Model (BCC-CSM): the main progress from CMIP5 to CMIP6. Geoscientific Model. Dev. 12, 1573–1600. doi: 10.5194/gmd-12-1573-2019

[B55] Xin X. Wu T. Zhang J. Yao J. Fang Y. (2020). Comparison of CMIP6 and CMIP5 simulations of precipitation in China and the East Asian summer monsoon. Int. J. Climatology 40, 6423–6440. doi: 10.1002/joc.6590

[B56] Xing Y. Chen M. Dao J. Lin L. Chen C. Chen Y. . (2024). Fine-root morphology of woody and herbaceous plants responds differently to altered precipitation: a meta-analysis. For. Ecol. Manage. 552, 121570. doi: 10.1016/j.foreco.2023.121570

[B57] Xu J. Wu Y. Wang S. Xu Y. Du C. Rohani E. R. . (2024). Predicting the potential distribution of *Arisaema heterophyllum* in China under current and future climate change based on ArcGIS and MaxEnt model. Plant Biosyst. 158, 1326–1334. doi: 10.1080/11263504.2024.2407813

[B58] Yadav B. Pandit D. L. Banjade D. Mehata D. K. Bhattarai S. Bhandari S. . (2024). Insights into the germplasm conservation and utilization: implications for sustainable agriculture and future crop improvement. Arch. Agric. Environ. Sci. 9, 180–193. doi: 10.26832/24566632.2024.0901026

[B59] Yan H. Feng L. Zhao Y. Feng L. Wu D. Zhu C. (2020). Prediction of the spatial distribution of *Alternanthera philoxeroides* in China based on ArcGIS and MaxEnt. Global Ecol. Conserv. 21, e00856. doi: 10.1016/j.gecco.2019.e00856

[B60] Yoon S. Lee W.-H. (2023). Application of true skill statistics as a practical method for quantitatively assessing CLIMEX performance. Ecol. Indic. 146, 109830. doi: 10.1016/j.ecolind.2022.109830

[B61] Zeng J. Li C. Liu J. Li Y. Hu Z. He M. . (2021). Ecological assessment of current and future *Pogostemon cablin* potential planting regions in China based on MaxEnt and ArcGIS models. J. Appl. Res. Medicinal Aromatic Plants 24, 100308. doi: 10.1016/j.jarmap.2021.100308

[B62] Zhang X. Zhai Y. Yuan J. Hu Y. (2019). New insights into *Paeoniaceae* used as medicinal plants in China. Sci. Rep. 9, 18469. doi: 10.1038/s41598-019-54863-y, PMID: 31804561 PMC6895042

[B63] Zhao D. Han C. Zhou C. Tao J. (2015). Shade ameliorates high temperature-induced inhibition of growth in herbaceous peony (*Paeonia lactiflora*). Int. J. Agric. Biol. 17, 911–919. doi: 10.17957/IJAB/15.0004

